# Detection of Significant Association Between Variants in Cannabinoid Receptor 1 Gene (*CNR1*) and Personality in African–American Population

**DOI:** 10.3389/fgene.2018.00199

**Published:** 2018-06-14

**Authors:** Yinghao Yao, Yi Xu, Junsheng Zhao, Yunlong Ma, Kunkai Su, Wenji Yuan, Jennie Z. Ma, Thomas J. Payne, Ming D. Li

**Affiliations:** ^1^State Key Laboratory for Diagnosis and Treatment of Infectious Diseases, The First Affiliated Hospital, Collaborative Innovation Center for Diagnosis and Treatment of Infectious Diseases, Zhejiang University School of Medicine, Hangzhou, China; ^2^Department of Public Health Sciences, University of Virginia, Charlottesville, VA, United States; ^3^Department of Otolaryngology and Communicative Sciences, University of Mississippi Medical Center, Jackson, MS, United States; ^4^Research Center for Air Pollution and Health, Zhejiang University, Hangzhou, China; ^5^Institute of Neuroimmune Pharmacology, Seton Hall University, South Orange, NJ, United States

**Keywords:** *CNR1*, SNP, African-Americans, NEO-FFI, personality traits

## Abstract

**Background:** Several studies have revealed significant associations between single nucleotide polymorphisms (SNPs) in the cannabinoid receptor 1 (*CNR1*) gene and a broad spectrum of psychiatric disorders such as major depressive disorder (MDD), attention deficit hyperactivity disorder (ADHD), and schizophrenia. Personality traits that are highly related to susceptibility to these conditions have been associated with the *CNR1* variants in subjects of Caucasian origin. However, there are no reported studies regarding the effects of *CNR1* polymorphisms on personality traits in the African-American (AA) population.

**Methods:** We performed an imputation-based association analysis for 26 *CNR1* variants with five dimensions of personality in 3,046 AAs.

**Results:** SNPs rs806372 and rs2180619 showed a significant association with extraversion after Bonferroni correction for multiple testing (*p* < 0.0019). Further, several extraversion-associated SNPs were significantly associated with conscientiousness, agreeableness, and openness. SNP priority score analysis indicated that SNPs rs806368, rs806371, and rs2180619 play a role in the modulation of personality and psychiatric conditions.

**Conclusion:** *CNR1* is important in determining personality traits in the AA population.

## Introduction

Personality traits can affect human behavior and predict health outcomes. They commonly are measured by the revised NEO Personality Inventory (NEO-PI-R) (Costa et al., 1992). The NEO-PI-R examines the following five factors: agreeableness, conscientiousness, extraversion, neuroticism, and openness (McCrae et al., 2005). These five dimensions, which account for nearly all of the differences in personality between individuals, have been linked to emotional stability, active motivation, and cognition (Terracciano et al., 2008b; DeYoung et al., 2010). Furthermore, the score for each personality trait has been used to predict many psychiatric disorders. For example, high scores of neuroticism, extraversion, and openness are considered indicators of bipolar disorder (Barnett et al., 2011), a high neuroticism score is associated with major depression disorder (MDD) and anxiety (Hettema et al., 2006), and a low conscientiousness score renders one prone to MDD (Kendler and Myers, 2010). In addition, substance abuse behaviors are related to personality. For example, tobacco smokers and cocaine and heroin users score high on neuroticism and low on conscientiousness (Terracciano and Costa, 2004; Terracciano et al., 2008a; Choi et al., 2017).

Family, twin, and adoption studies have demonstrated that personality traits are highly heritable, with an estimated heritability ranging from 40 to 60% (Horn et al., 1976; Floderus-Myrhed et al., 1980; Jang et al., 1996). Recent advances in genome-wide association studies (GWAS) have provided a genetic map for various personality traits (Amin et al., 2012, 2013; de Moor et al., 2012; Kim et al., 2013, 2015; Genetics of Personality Consortium et al., 2015; Okbay et al., 2016; Smith et al., 2016; van den Berg et al., 2016; Lo et al., 2017; Luciano et al., 2018). Of them, neuroticism and extraversion are the two best-researched traits, uncovering a number of related loci in independent samples (Calboli et al., 2010; Genetics of Personality Consortium et al., 2015; Okbay et al., 2016; Smith et al., 2016; van den Berg et al., 2016; Luciano et al., 2018). However, although the most significant loci for several personality traits have been identified, only a small fraction of heritability could be explained by these top hits, indicating that single nucleotide polymorphisms (SNPs) with effect sizes well below genome-wide significance likely account for the “missing heritability” (Manolio et al., 2009; Yang et al., 2010). Thus, the genetic architecture of personality requires further investigation, especially for those loci below the commonly accepted GWAS threshold (Boyle et al., 2017).

Although it has not been revealed to be significant at the genome-wide significant level by any GWAS, cannabinoid receptor 1 (*CNR1*) is a plausible candidate gene for certain personality traits. Juhasz et al. (2009a) first reported a significant association of *CNR1* with personality traits in a Caucasian population. By genotyping seven tag SNPs plus three predictive expression quantitative loci, they detected several haplotypes that were significantly associated with neuroticism and agreeableness, and substantial phenotypic variances were explained by the *CNR1* gene variants. Furthermore, a recent study showed an association between *CNR1* rs7766029 and neuroticism (Aleksandrova et al., 2012). In addition, a genome-wide linkage study based on the Erasmus Rucphen Family sample (Pardo et al., 2005) detected a significant linkage signal for extraversion at a genomic region near *CNR1* (Amin et al., 2013). The protein encoded by this gene belongs to the G-protein receptor family and modulates neurotransmitter release by coupling with a decreased intracellular cAMP concentration (Elphick and Egertova, 2001). CB_1_ receptors are densely expressed in the central nervous system and act as neuromodulators to inhibit the release of glutamate and GABA (Elphick and Egertova, 2001).

Several genetic association studies have implicated the *CNR1* in the risk of several psychiatric disorders (Juhasz et al., 2009a,b, 2017; Lazary et al., 2009). It has been reported that genetic variants in the promoter region of *CNR1* and serotonin transporter gene (*SLC6A4*) interactively increase the risk of high anxiety scores (Lazary et al., 2009), and interaction of *CNR1* variants with recent negative life events is considered to be an important risk factor for development of depression symptoms (Juhasz et al., 2009a) and migraine (Juhasz et al., 2017). Furthermore, additional data indicate a nominal association between *CNR1* variants and metabolic syndrome in patients with schizophrenia (Ujike et al., 2002; Martinez-Gras et al., 2006; Yu et al., 2013) and attention deficit hyperactivity disorder (ADHD) (Lu et al., 2008).

Given that the *CNR1* gene has been implicated in the pathology of a wide range of psychiatric disorders, examining its effects on personality traits is of great interest, as they are regarded as risk factors for these disorders. Although a significant association of the gene with personality has been uncovered in Caucasian populations, little is known about non-Caucasian populations such as persons of African origin. Thus, in the present study, we focused on determining whether there exists any significant association between the *CNR1* variants and five dimensions of personality in the African-American (AA) population.

## Materials and Methods

### Subjects

The 3,046 AA subjects used in this study were recruited during 2005–2011 as part of the Mid-South Tobacco Case-Control (MSTCC) study (Yang et al., 2015). Of note, all AA samples used in this study were recruited from the Jackson area of Mississippi, where residents do not relocate or migrate as often as residents of other large cities within the United States, implying that they are relatively more homogeneous. These subjects aged 18 or older were all biologically unrelated. The NEO Five-Factor Inventory (NEO-FFI) questionnaire (McCrae et al., 2005) was used to assess the five dimensions of personality. The characteristics of the MSTCC AA sample are shown in **Table 1**. After a detailed explanation of the project was provided to potential participants, informed written consent was obtained. The Institutional Review Boards of the University of Virginia and University of Mississippi Medical Center approved this study.

**Table 1:** Characteristics of the five dimensions of personality traits.

Sample size	3,046
Female, *N* (%)	1,399 (45.92)
Age in years, mean (SD)	42.73 (±13.54)
Age range (years)	18–88
Agreeableness score, mean (SD)	37.26 (±3.11)
Extraversion score, mean (SD)	39.84 (±2.68)
Openness score, mean (SD)	43.66 (±2.97)
Conscientiousness score, mean (SD)	38.83 (±2.15)
Neuroticism score, mean (SD)	33.34 (±2.97)

### Questionnaires

The background information collected from participants included age, sex, ethnicity, education, and smoking history. Sixty items of NEO-FFI (Costa and McCrae, 1997) were used to define the five personality dimensions of agreeableness, extraversion, openness to experience, conscientiousness, and neuroticism (12 items per factor). Items were answered on a 5-point Likert-type scale ranging from *strongly disagree* (score = 0) to *strongly agree* (score = 4).

### Genotyping and Imputation

For all participants, peripheral blood DNA was extracted using Qiagen DNA isolation kits. Genotyping was conducted by HumanExome BeadChip (v1.1) according to the manufacturer’s instruction (Illumina Inc., San Diego, CA, United States). Genotypes were called with Illumina GenomeStudio software. Following genotyping, we conducted whole-genome imputation using IMPUTE2 (Howie et al., 2012) in 5-Mb chunks after prephasing with SHAPEIT2 (Delaneau et al., 2013). The haplotype panels released by the 1000 Genome Project (1000 Genomes Project Consortium et al., 2010) Phase3^1^ (October 2014) were used as a reference.

**FIGURE 1:**
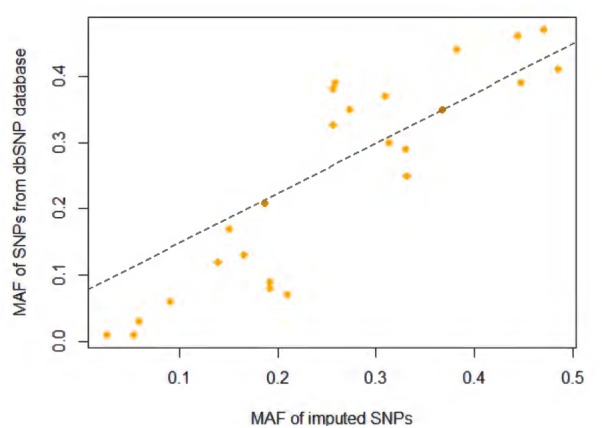
Minor allele frequency (MAF) comparison between imputed SNPs in current study and SNPs in 1000 Genome Project African population.

For this study, SNPs located in the start and end positions of *CNR1* plus 20-Kb regions on both sides were extracted (Chr6: 88829583-88896063, NCBI build 37). As shown in Supplementary Figure S1, most SNPs have a low minor allele frequency (MAF) (<0.01), and any SNP with a MAF of less than 0.01 were removed from the following analysis. The recommended cutoff of 0.3 for the “info” metric was used to filter poorly imputed variations (Marchini and Howie, 2010). After quality control, 26 imputed SNPs were included in the association analysis (Supplementary Table S1). Finally, we compared the MAF between imputed SNPs in this study and corresponding variants in 1000 Genome Project African population (**Figure 1**) and found them to be quite consistency of each other. A detailed genotyping information for all samples used in the study is provided in Supplementary Data Sheet S1.

### Individual SNP-Based Association Analysis

We used the PLINK program (v. 1.07) (Purcell et al., 2007) to perform linear regression for each personality trait under an additive genetic model with age and sex included as covariates. Briefly, fcGENE (Roshyara and Scholz, 2014) was fitted into imputation workflow to convert genotype probability data to the PLINK file format with a tolerant cutoff value of 0.5. Standard quality controls for converted genotype data were implemented by PLINK.

To account for the probabilistic nature of imputed genotypes, frequentist tests, implemented by SNPTEST (v2.5.2) (Marchini et al., 2007), were used to evaluate the effects of *CNR1* variants on personality traits. We applied two types of frequentist tests (Marchini and Howie, 2010). The Score test uses genotype likelihood to perform association tests in the way that each possible genotype is weighted by its imputation probability. The Expected test treats the expected allele count (also called allele dosage) as an independent variable and phenotype as dependent variable and then relates them by regression analysis. Sex and age were included as covariates in the association analysis. Based on the design for a candidate gene study, we adopted a relatively conservative Bonferroni correction for 26 SNPs, with a *p*-value less than 0.0019 (0.05/26) being considered statistically significant. Quanto v1.2.4^2^ was employed to estimate the power of this study.

### SNP Priority Score

For the identified SNPs, we calculated SNP priority scores by integrating evidence from single marker statistics, functional annotation, and previous studies. First, SNPs shown to be associated with any personality trait in this study were each assigned one point, which is accumulated if more than one trait is marked. Second, each SNP annotated by HaploReg v4.1 (Ward and Kellis, 2012) as exonic, splicing, 5′-UTR, 3′-UTR, promoter or enhancer was assigned one point. Third, SNPs reported to be associated with personality traits, psychiatric disorders, and emotional behaviors in other studies from the literature were highlighted again. Finally, we added all the scores as the SNP priority score as described previously (Imamura et al., 2016).

## Results

**FIGURE 2:**
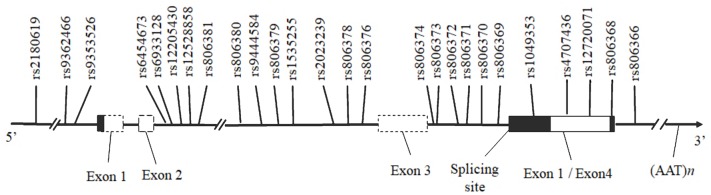
Schematic diagram of the human *CNR1* and the positions of the SNPs investigated. Horizontal black arrows indicate the direction of transcription. The white bar represents the exonic region according to the National Center for Biotechnology Information (NCBI) and the University of California at Santa Cruz Browser (UCSC). The gray region within the white bar corresponds to the alternative splicing coding region, and the untranslated region is shown in black.

**Table 1** and Supplementary Figure S2 present the score statistics and distributions of five personality dimensions for all participants. All five dimensions showed a pattern of normal distribution. SNPs included in this report are shown in Supplementary Table S1, which spans from the 5′ promoter to 3′ downstream of the *CNR1* gene region (**Figure 2**). We observed a very high consistency of allele frequency distribution between our imputed SNPs and those deposited in the dbSNP database (1000 Genomes Project Consortium et al., 2012) (*R*^2^ = 0.79, *p* = 9.13 × 10^-10^; **Figure 1**).

### Association of Individual SNPs With Personality

Detailed association analysis of individual SNPs with five dimensions of personality is shown in Supplementary Tables S2–S6. Overall, we found that the *p* values estimated by the Score and Expected tests were highly concordant (*R*^2^ > 0.99), which is in line with a previous report that these two approaches show a great approximation when the effect size of risk alleles is small (Marchini and Howie, 2010). On the other hand, the frequentist test appeared to detect more significant results than PLINK did, indicating that genotype uncertainty represents a major factor with the imputed data.

For the case-control study on quantitative traits under the hypothesis of a gene-only model that directly relates phenotype to genotype, we have 94.97% power to detect a polymorphism (with an allele frequency > 1%) that could explain 1% of the variance of the traits at the 0.0001 two-tailed significance level with a sample size of 3,046, as used in this study.

For extraversion, 13 SNPs showed nominal significance (*P* < 0.05; see Supplementary Table S2). Of these significant SNPs, the association of rs806368 (*p* = 1.79 × 10^-4^), rs806370 (*p* = 2.68 × 10^-4^), rs806372 (*p* = 2.66 × 10^-4^), and rs2180619 (*p* = 1.53 × 10^-3^) remained significant after correction for multiple testing. To identify the potential significance-driven signal(s), we controlled the most significant SNP (rs806368) for the other three SNPs under investigation. A residual association was observed at SNP rs2180619 (*p* = 0.017), indicating that there might exist independent signal(s) within this region. Notably, all the significant SNPs showed negative associations with extraversion, indicating the *CNR1* gene has a regulatory effect on the extraversion score.

For conscientiousness, the strongest association was detected on rs2180619 (*p* = 3.01 × 10^-4^) in the 5′ promoter region of *CNR1* (**Table 2** and Supplementary Table S3), an important region likely involved in regulating mRNA expression of the gene (Zhang et al., 2004). The other two SNPs significantly associated with conscientiousness were rs9353526 (*p* = 9.97 × 10^-4^) and rs9362466 (*p* = 1.36 × 10^-3^), both of which are located in the upstream region of *CNR1*. After conditioning on rs2180619, we found no evidence of any association signal for these two SNPs.

**Table 2:** Identified SNPs associated with dimensions of personality.

SNP ID	Position	Minor allele	MA F	Extraversion		Conscientiousness		Agreeableness		Openness		Neuroticism		SNP location s
				*P*-Value	Beta	*P*-Value	Beta	*P*-Value	Beta	*P*-Value	Beta	*P*-Value	Beta	
rs806368	88850100	C	0.210	**1.79E-03**	-0.152	0.294	-0.051	0.155	-0.069	*0.021*	-0.113	0.484	-0.034	3-UTR
rs12720071	88851181	C	0.151	*0.028*	0.133	0.073	0.109	0.660	-0.027	0.466	0.044	0.444	0.046	3-UTR
rs806369	88856178	T	0.139	*0.016*	-0.166	*0.013*	-0.170	0.426	-0.054	0.193	-0.089	0.611	0.035	Intronic
rs806370	88856331	T	0.192	**2.68E-04**	-0.184	0.150	-0.073	0.256	-0.057	*0.017*	-0.120	0.402	-0.042	Intronic
rs806371	88856363	G	0.331	*0.013*	-0.109	*0.044*	-0.088	0.645	-0.020	*0.018*	-0.104	0.619	-0.022	Intronic
rs806372	88856563	C	0.192	**2.66E-04**	-0.184	0.151	-0.073	0.256	-0.057	*0.017*	-0.121	0.401	-0.043	Intronic
rs806376	88858648	T	0.309	*0.002*	-0.132	0.174	-0.059	0.695	-0.017	0.084	-0.075	0.849	0.008	Intronic
rs806379	88861267	A	0.471	0.037	-0.087	0.851	-0.008	0.703	-0.016	0.432	-0.033	0.874	0.007	Intronic
rs9444584	88862559	C	0.444	0.007	-0.108	0.351	-0.037	0.805	-0.010	*0.023*	-0.091	0.793	0.010	Intronic
rs806381	88865901	G	0.330	0.135	0.071	0.330	0.046	0.783	0.013	0.158	0.067	0.905	0.006	Intronic
rs6454673	88871049	A	0.313	0.032	0.104	0.256	0.055	0.563	0.028	0.212	0.060	0.834	-0.010	Intronic
rs9353526	88876576	T	0.256	*0.002*	-0.153	**9.97E-04**	-0.164	0.058	-0.094	0.060	-0.094	0.309	0.051	Upstream
rs9362466	88876581	T	0.258	*0.003*	-0.146	**1.36E-03**	-0.159	0.052	-0.096	0.072	-0.089	0.342	0.047	Upstream
rs2180619	88877952	A	0.256	**1.53E-03**	-0.158	**3.01E-04**	-0.180	*0.045*	-0.100	0.080	-0.087	0.266	0.055	Promoter

*MAF, minor allele frequency; Func Annot, functional annotation; nominal significances are given in italics (*p* < 0.05); Bonferroni correction significances are given in bold (*p* < 1.9E-03)*.

For openness, five SNPs showed nominal significance (*p* < 0.05, Supplementary Table S4). Of them, SNPs rs806368, rs806370, and rs806372 also showed significantly association with extraversion (**Table 2**). It is worthwhile to note that SNP rs806368 has been reported to be significantly associated with openness in a sample with Caucasian origins (Juhasz et al., 2009a).

For agreeableness, we found four marginally associated loci, with the SNP rs2180619 being the strongest (*p* = 0.007, Supplementary Table S5). Importantly, these SNPs were detected only with PLINK, but not with SNPTEST, indicating a potentially less reliable result generated from missing genotypes. However, the agreeableness-associated SNPs were linked to extraversion (**Table 2**). For neuroticism, we did not observe any significance for the SNPs investigated (Supplementary Table S6).

### Priority of Important SNPs Implicated in Personality Traits and Neuropsychiatry Disorders

**FIGURE 3:**
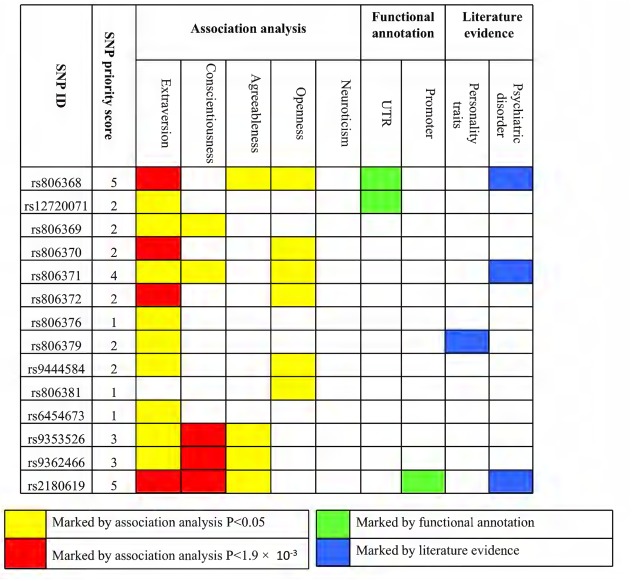
Prioritized SNPs with current and previous evidence. Filled orange boxes indicate evidence from association analysis with nominal significance (*P* < 0.05). Filled red boxes indicate evidence from association analysis with multiple testing correction significance (*P* < 0.0019). Filled green boxes indicate evidence from functional annotation. Filled blue boxes indicate evidence from the published literature. SNP priority scores were counted by adding each piece of evidence.

Through integrating multiple lines of evidence for associated SNPs and calculating SNP priority score, we revealed that variants in *CNR1* contributed to the genetic component of personality and their related psychiatric disorders, particularly for rs806368, rs806371, and rs2180619, whose priority scores equaled or were greater than 4 (range 1–9; **Figure 3** and Supplementary Table S7).

## Discussion

In the present study, we identified several variants in *CNR1* showing significant associations with various dimensions of personality in an AA sample, especially for extraversion, for which four SNPs were identified. Importantly, SNPs associated with the other four dimensions of personality were all associated with extraversion. Considering that variants in *CNR1* have been reported to be associated with an increased risk of mental health conditions, these data indicate the presence of genetic influences shared by personality traits and psychiatric disorders (Hettema et al., 2006; Kendler and Myers, 2010).

Our study might benefit greatly from the AA samples used in this study compared with those reported GWAS studies on various personality traits (de Moor et al., 2012; Smith et al., 2016), where samples with multiple ethnic origins, locations, or studies designed for different purposes were included. By using two approaches to analyze the same imputed dataset, we found that the frequentist test performed better than the regression model. When compared with PLINK in the context of genotype uncertainty, the frequentist tests, including the Score and Expected tests, improved statistical significance. One of the possible explanations for this result is that SNPTEST fully accounts for the uncertainty in imputed genotypes by weighting the imputation probability (Score test) or by calculating the allele dosage (Expected test). Conversely, the PLINK-based regression analysis uses only imputed genotypes that have a posterior probability above some threshold, which might lead to both false-positive findings and loss of power (Marchini and Howie, 2010). By implementing a gene-only model-based power analysis, we believe that the current genetic study is able to detect significant effects of *CNR1* variants on personality traits in this valuable AA sample.

The relations between *CNR1* and personality traits as well as other psychiatric disorders, such as depression and anxiety, have been established by Juhasz et al. (2009a), where 10 SNPs in *CNR1* were genotyped, but they failed to detect any significant association with any personality trait at the individual SNP level in 1,269 participants. Interestingly, the haplotype trend regression analysis revealed significant associations with neuroticism and agreeableness after correction for multiple testing. Furthermore, those investigators detected SNP-by-SNP interactions for rs806379 with several other SNPs on both neuroticism and agreeableness. Another genetic association study revealed the association of rs7766029 with neuroticism (Aleksandrova et al., 2012).

Our individual SNP-based association analysis indicated significant association of *CNR1* variants with extraversion in AAs. Rather than selecting haplotype-tagged SNPs, we imputed SNPs within the region of interest according to a high-density reference panel. An established statistical method was used in this study, which takes the genotype uncertainty into account and appeared to be more sensitive than PLINK in terms of our imputed data. Although almost all the investigated SNPs in the study reported by Juhasz et al. (2009a) were included in our analysis (except for rs7766029 because of the lack of reliable imputation quality), comparable results were not obtained in the two studies. We detected a positive association only between rs806368 and openness regardless of the genetic model. Such divergent results might be explained by the following factors: (Costa et al., 1992) different genetic models were used in these two studies. We detected single marker associations with the additive model, whereas Juhasz et al. (2009a) reported their results from three models (additive, dominant, and recessive); (McCrae et al., 2005) different questionnaires were used in these two studies. Juhasz et al. (2009a) used the Big Five Inventory (BFI-44) to assess personality, whereas we used the NEO Personality Inventory. Although there exist excellent correlations between the BFI-44 Inventory and NEO, a slight discrepancy might arise in phenotypic assessments; and (DeYoung et al., 2010) population stratification: we observed differential allele frequencies between AAs and European-American (EA) populations (Supplementary Table S8). For example, the allele frequency of rs1049359 is rarer in AAs (MAF = 0.029) than in EAs (MAF = 0.258).

The extraversion-associated SNP rs806368 remained significant after Bonferroni correction. In addition, this SNP showed a marginal association with both agreeableness and openness (**Table 2**). Rs806368 is located in the 3′-UTR region of *CNR1*. A strong association between rs306368 and impulsivity has been reported for the Southwest California Indians (also known as Mission Indians) (Ehlers et al., 2007). Impulsivity is considered as a facet of personality and acts as a major component of various psychiatric diseases such as ADHD (Nigg, 2001), bipolar disorder (Henry et al., 2001), and antisocial personality disorder (Horn et al., 2003). The study reported by Eysenck (1967) indicates that extraversion is a combination of impulsiveness and sociability, which is consistent with the genetic findings that rs806368 is associated with extraversion and impulsivity.

A pharmacogenomics study highlighted the significant role of rs806371 in both MDD etiology and the clinical response to citalopram treatment (Mitjans et al., 2013). Mitjans et al. (2013) reported a higher frequency of rs806371 G carriers in MDD patients with melancholia and psychotic symptoms than in controls. Moreover, the C allele carriers of rs806371 demonstrate increased remission status of MD symptoms after 12 weeks of treatment with citalopram (Mitjans et al., 2013).

Another interesting SNP revealed by this study is rs2180619, located in the promoter region of *CNR1*, which is not only significantly associated with both extraversion and conscientiousness, but also nominally with agreeableness (**Table 2**). By genotyping 706 individuals for the 5′-HTTLPR in the *SLC6A4* and four SNPs in *CNR1*, Lazary et al. (2009) showed that the risk of anxiety was 4.6-fold greater in individuals with genotypes G/G for rs2180619 and S/S for 5-HTTLPR compared with other genotypes. Furthermore, Heitland et al. (2012) found that individuals with genotypes G/G or G/A for rs2180619 showed robust extinction of fear compared with individuals with the A/A genotype. Interestingly, Mroczkowski et al. (2016) found that a high neuroticism score significantly increases the risk of three anxiety disorders (agoraphobia, panic disorder, and social phobia), and extraversion and conscientiousness were negatively associated with separation anxiety disorder and social phobia. Although these relationships were constructed in individuals with obsessive compulsive disorder, the significances survived after adjusting for age at onset of this disorder (Mroczkowski et al., 2016).

Relationships among personality traits are worthy of consideration. For example, extraversion and openness shared genetic overlap in the context of rs806368, rs806370, rs806371, rs806372, and rs9444584; extraversion, conscientiousness, and agreeableness were highly related for rs9353526, rs9362466, and rs2180619 (**Table 2**). We further found the direction of effects for associated SNPs were opposite between neuroticism and the other four personality traits. It was reported recently that neuroticism shows a negative genetic correlation with the other four personality traits (Lo et al., 2017). Taken together, the multiple dimensions of personality-associated *CNR1* variants support the concept that the presence of shared genetic factors contributes to the correlation among personalities (Lo et al., 2017), suggesting a fundamental role of *CNR1* in personality formation.

Dysfunction of *CNR1* may result in neuropsychological disturbances, leading to psychiatric disorders. Martin et al. (2002) first used CB1 knockout mice to evaluate the potential role of *CNR1* in emotional behavior (Ledent et al., 1999). By using a light/dark box, they observed a significant decrease in the number of entries and in the time spent in the lit compartment for CB1 knockout mice, indicating that these mice present an anxiogenic-like response. By using a chronic unpredictable mild stress model (CMS) to simulate anhedonia, the CB1 knockout mice exhibited greater anhedonia, a core symptom of depression and defining feature of melancholia (Klonsky, 2008; Battle, 2013). More recently, Bowers and Ressler (2016) administered *Cnr1* antagonist SR141716A to C57BL/6J mice, after which both male and female adult mice were sensitive to *Cnr1* antagonist-mediated increases in anxiety-like behavior, thus supporting the protective role of *CNR1* against the development of anxiety disorder. These studies confirmed that dysfunction of the CB1 receptor changes several behavioral responses and increases susceptibility to emotional disorders, such as anxiety, depression, and aggressiveness.

There are limitations associated with this study. First, the SNPs investigated were generated primarily by imputation. Although we have great confidence in the accuracy of our imputed genotypes, as demonstrated in the literature, including our own work (Marchini and Howie, 2010; Howie et al., 2011), individual genotyping of variants in *CNR1* is desirable in future studies to support the conclusions about the association of *CNR1* variants with personality in AAs. Second, our imputation-based SNP selections provide a fine-mapping of *CNR1*, but the SNPs examined could not accurately tag the causal genetic variants. Last but not least, the detected associations of variants in *CNR1* with various personality traits require to be replicated with independent AA samples. However, such type of AA samples with appropriate phenotypes is unavailable to us.

## Conclusion

This is the first study to demonstrate the significant effects of *CNR1* polymorphisms on personality traits in AAs. The SNPs identified in this study revealed significant associations with multiple personality traits, indicating the fundamental role of *CNR1* variants in the expression of personality. Furthermore, previous publications have related our identified personality-associated SNPs with psychiatric disorders, which provides additional evidence supporting the presence of genetic correlation between personalities and mental health conditions. Understanding these relationships may reveal opportunities to prevent and treat neuropsychiatric disorders.

## Author Contributions

JM, TP, and ML participated in clinical data collection. YY, JZ, WY, KS, and YM performed the laboratory experiments. YY, YX, KS, and YM conducted the data analysis. YY, TP, and ML participated in writing of the manuscript. ML conceived the study and involved in every step of the work. All authors approved the final draft of the paper.

## Conflict of Interest Statement

The authors declare that the research was conducted in the absence of any commercial or financial relationships that could be construed as a potential conflict of interest.

## Footnotes

1 http://mathgen.stats.ox.ac.uk/impute/1000GP_Phase3.html

2 http://biostats.usc.edu/Quanto.html
