# Supplementary material for: Detection of Significant Association Between Variants in Cannabinoid Receptor 1 Gene (CNR1) and Personality in African–American Population
Source: Front Genet. 2018 Jun 14;9:199. doi: 10.3389/fgene.2018.00199 (PMC6010580; doi:10.3389/fgene.2018.00199)
Supplement: Supplementary file 1 [file Presentation_1.pdf]

Detection of significant association between variants in cannabinoid receptor 1 gene (*CNR1*) and personality in African-American population

Yinghao Yao, Yi Xu, Junsheng Zhao, Yunlong Ma, Kunkai Su,
Wenji Yuan, Jennie Z Ma, Thomas J Payne, and Ming D. Li

Supplemental Figures

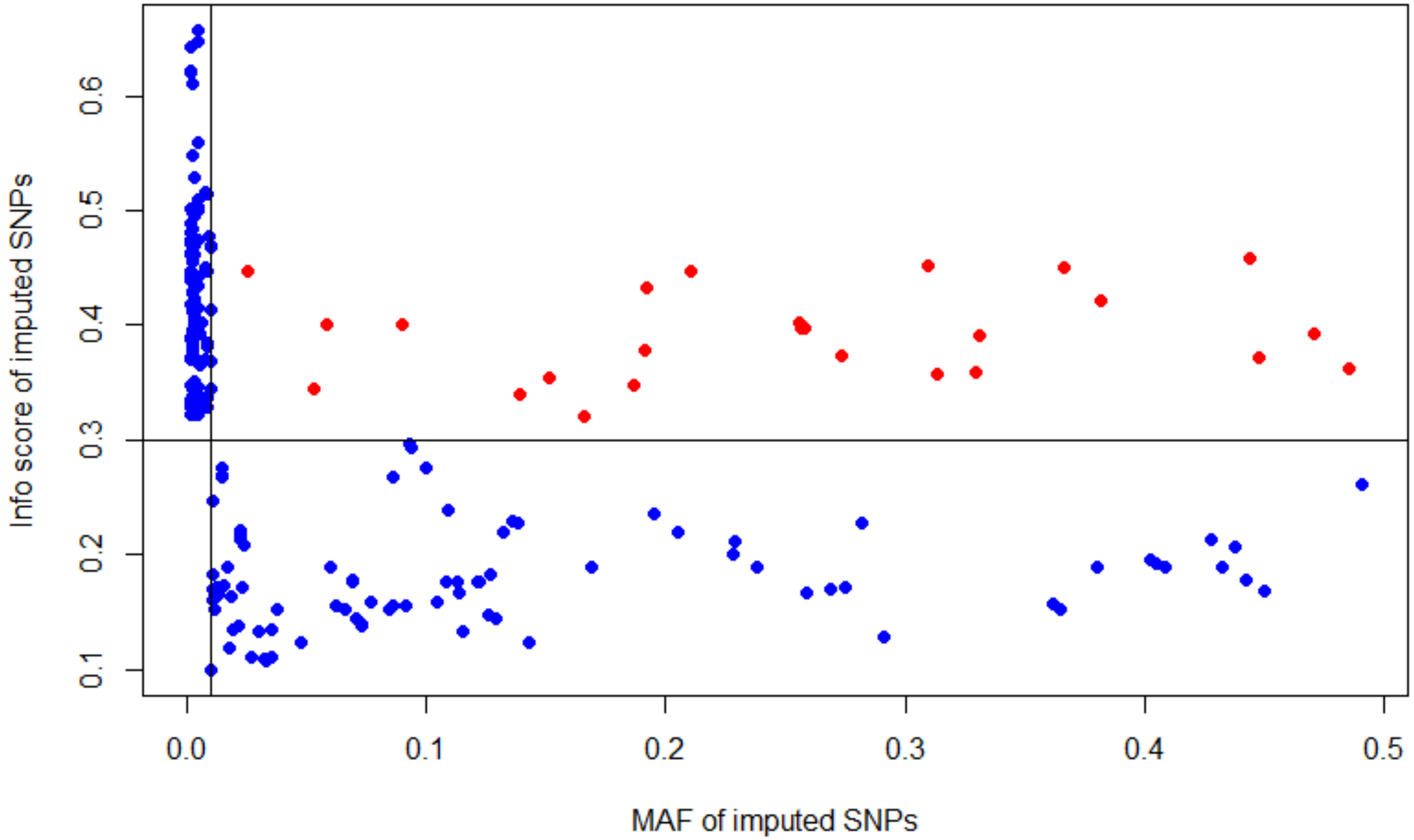

Supplemental Figure S1: A plot of MAF and "info" metric for 273 imputed SNPs. Red dots indicate SNPs remained for association analysis and blue dots indicate that those SNPs were removed from further analysis due to their low MAF and 'info' scores.

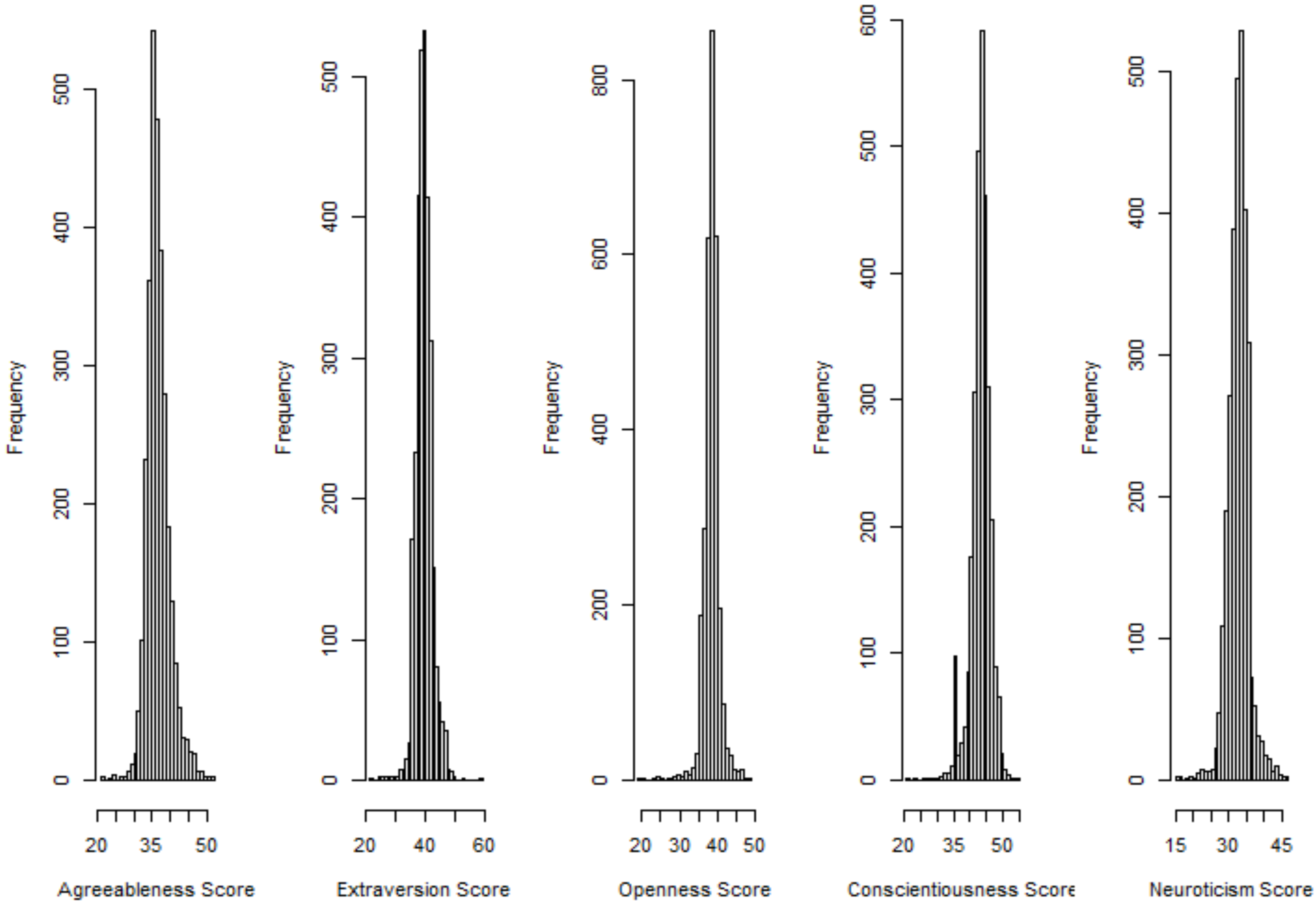

Supplemental Figure S2: The distributions of personality scores of five dimensions in the African-American sample used in this study.

Supplemental Table S1: Summary information of the investigated SNPs

SNP ID	Position	Ref. allele	Alt. allele	Info score	Imputed MAF	dbSNP AFR MAF	SNP Locations
rs806366	88847589	C	T	0.312	0.186	0.208	downstream
rs806368	88850100	T	C	0.447	0.210	0.076	3-UTR
rs12720071	88851181	T	C	0.355	0.151	0.177	3-UTR
rs4707436	88851751	G	A	0.421	0.382	0.417	3-UTR
rs1049353	88853635	C	T	0.401	0.058	0.029	Exonic
rs806369	88856178	T	C	0.340	0.139	0.124	Intronic
rs806370	88856331	C	T	0.433	0.192	0.077	Intronic
rs806371	88856363	T	G	0.391	0.331	0.254	Intronic
rs806372	88856563	G	C	0.433	0.192	0.077	Intronic
rs806373	88856916	T	A	0.372	0.448	0.428	Intronic
rs806374	88857320	T	C	0.363	0.485	0.417	Intronic
rs806376	88858648	T	C	0.452	0.309	0.314	Intronic
rs806378	88859551	C	T	0.400	0.090	0.058	Intronic
rs2023239	88860482	T	C	0.450	0.367	0.368	Intronic
rs1535255	88861208	T	G	0.373	0.273	0.372	Intronic
rs806379	88861267	A	T	0.392	0.471	0.442	Intronic
rs9444584	88862559	C	T	0.459	0.444	0.408	Intronic
rs806380	88864653	A	G	0.378	0.191	0.080	Intronic
rs806381	88865901	A	G	0.359	0.330	0.303	Intronic
rs12528858	88867488	A	G	0.447	0.025	0.010	Intronic
rs12205430	88867925	T	C	0.344	0.053	0.010	Intronic
rs6933128	88868830	C	A	0.320	0.165	0.140	Intronic
rs6454673	88871049	G	A	0.357	0.313	0.332	Intronic
rs9353526	88876576	G	T	0.397	0.256	0.323	Upstream
rs9362466	88876581	C	T	0.398	0.258	0.324	Upstream
rs2180619	88877952	G	A	0.365	0.256	0.326	Promoter

Notes: (1) Ref allele = reference allele; Alt allele = alternate allele; Imputed MAF= imputed minor allele frequency; dbSNP AFR MAF = minor allele frequency derived from dbSNP database AFR population by 1000 Genomes.

Supplemental Table S2: Statistical results for association analysis with Extraversion

SNP ID	Position	Minor allele	MAF	SNPTEST				PLINK	
				Expected P	Expected Beta	Score P	Score Beta	Plink P	Plink Beta
rs806366	88847589	C	0.186	0.195	-0.076	0.211	-0.071	0.342	-0.117
rs806368	88850100	C	0.210	**1.79E-03**	-0.152	*0.004*	-0.131	*0.047*	-0.204
rs12720071	88851181	C	0.151	*0.028*	0.133	*0.027*	0.135	0.104	0.221
rs4707436	88851751	A	0.382	0.209	0.052	0.222	0.049	0.361	0.077
rs1049353	88853635	T	0.058	0.940	-0.007	0.946	-0.006	0.975	0.007
rs806369	88856178	T	0.139	*0.016*	-0.166	*0.019*	-0.157	0.217	-0.184
rs806370	88856331	T	0.192	**2.68E-04**	-0.184	**6.62E-04**	-0.161	*0.016*	-0.261
rs806371	88856363	G	0.331	*0.013*	-0.109	*0.017*	-0.101	*0.029*	-0.194
rs806372	88856563	C	0.192	**2.66E-04**	-0.184	**6.58E-04**	-0.161	*0.015*	-0.264
rs806373	88856916	A	0.448	0.294	-0.045	0.305	-0.043	0.570	-0.052
rs806374	88857320	C	0.485	0.481	-0.030	0.487	-0.029	0.711	-0.034
rs806376	88858648	T	0.309	*0.002*	-0.132	*0.003*	-0.125	*0.009*	-0.228
rs806378	88859551	T	0.090	0.869	-0.012	0.874	-0.011	0.356	0.157
rs2023239	88860482	C	0.367	0.253	0.046	0.263	0.044	0.137	0.126
rs1535255	88861208	G	0.273	0.636	0.023	0.642	0.022	0.623	0.049
rs806379	88861267	A	0.471	*0.037*	-0.087	*0.040*	-0.085	0.059	-0.164
rs9444584	88862559	C	0.444	*0.007*	-0.108	*0.008*	-0.104	*0.005*	-0.221
rs806380	88864653	G	0.191	0.805	-0.014	0.804	-0.014	0.557	-0.065
rs806381	88865901	G	0.330	0.135	0.071	0.142	0.068	0.074	0.170
rs12528858	88867488	G	0.025	0.786	0.035	0.800	0.031	0.072	0.572
rs12205430	88867925	C	0.053	0.086	-0.176	0.102	-0.159	0.281	-0.254
rs6933128	88868830	A	0.165	0.293	0.067	0.273	0.072	0.243	0.142
rs6454673	88871049	A	0.313	*0.032*	0.104	*0.032*	0.103	*0.029*	0.209
rs9353526	88876576	T	0.256	*0.002*	-0.153	*0.003*	-0.144	*0.002*	-0.306
rs9362466	88876581	T	0.258	*0.003*	-0.146	*0.004*	-0.137	*0.002*	-0.301
rs2180619	88877952	A	0.256	**1.53E-03**	-0.158	*0.002*	-0.147	**1.70E-03**	-0.309

Notes: (1) MAF = minor allele frequency; (2) Nominal significances given in italic ($p < 0.05$); (3) Bonferroni correction significances given in bold (p < 1.9E-03).

Supplemental Table S3: Statistical results for association analysis with Conscientiousness

SNP ID	Position	Minor allele	MAF	SNPTEST				PLINK	
				Expected P	Expected Beta	Score P	Score Beta	Plink P	Plink Beta
rs806366	88847589	C	0.186	0.303	-0.061	0.287	-0.064	0.742	-0.045
rs806368	88850100	C	0.210	0.294	-0.051	0.306	-0.049	0.605	-0.059
rs12720071	88851181	C	0.151	0.073	0.109	0.070	0.111	0.596	0.080
rs4707436	88851751	A	0.382	0.959	0.002	0.958	0.002	0.519	0.060
rs1049353	88853635	T	0.058	0.239	-0.108	0.285	-0.089	0.400	-0.200
rs806369	88856178	T	0.139	*0.013*	-0.170	*7.94E-03*	-0.195	0.147	0.239
rs806370	88856331	T	0.192	0.150	-0.073	0.154	-0.071	0.757	-0.037
rs806371	88856363	G	0.331	*0.044*	-0.088	*0.039*	-0.093	0.162	-0.137
rs806372	88856563	C	0.192	0.151	-0.073	0.155	-0.071	0.709	-0.045
rs806373	88856916	A	0.448	0.402	-0.036	0.393	-0.037	0.586	0.054
rs806374	88857320	C	0.485	0.246	-0.050	0.233	-0.052	0.946	-0.007
rs806376	88858648	T	0.309	0.174	-0.059	0.167	-0.060	0.401	-0.080
rs806378	88859551	T	0.090	0.962	0.004	0.962	0.004	0.920	-0.019
rs2023239	88860482	C	0.367	0.896	0.005	0.894	0.005	0.122	0.144
rs1535255	88861208	G	0.273	0.373	-0.043	0.353	-0.046	0.915	-0.012
rs806379	88861267	A	0.471	0.851	-0.008	0.847	-0.008	0.612	-0.048
rs9444584	88862559	C	0.444	0.351	-0.037	0.345	-0.038	0.298	-0.091
rs806380	88864653	G	0.191	0.471	-0.040	0.456	-0.043	0.397	-0.105
rs806381	88865901	G	0.330	0.330	0.046	0.316	0.049	0.159	0.147
rs12528858	88867488	G	0.025	0.763	0.039	0.770	0.037	0.140	0.521
rs12205430	88867925	C	0.053	0.279	-0.111	0.236	-0.132	0.584	-0.143
rs6933128	88868830	A	0.165	0.394	0.054	0.342	0.067	0.247	0.155
rs6454673	88871049	A	0.313	0.256	0.055	0.238	0.059	0.218	0.130
rs9353526	88876576	T	0.256	**9.97E-04**	-0.164	**7.08E-04**	-0.173	**9.66E-05**	-0.424
rs9362466	88876581	T	0.258	**1.36E-03**	-0.159	**9.99E-04**	-0.168	**1.74E-04**	-0.408

rs2180619	88877952	A	0.256	**3.01E-04**	-0.180	**2.20E-04**	-0.188	**1.84E-05**	-0.464

Notes: (1) MAF = minor allele frequency; (2) Nominal significances given in italic ($p < 0.05$); (3) Bonferroni correction significances given in bold (p < 1.9E-03).

Supplemental Table S4: Statistical results for association analysis with Openness

		Minor		SNPTEST				PLINK	
SNP ID	Position	allele	MAF	Expected P	Expected Beta	Score P	Score Beta	Plink P	Plink Beta
rs806366	88847589	C	0.186	0.573	0.033	0.571	0.033	0.936	-0.008
rs806368	88850100	C	0.210	*0.021*	-0.113	*0.028*	-0.102	0.076	-0.147
rs12720071	88851181	C	0.151	0.466	0.044	0.464	0.044	0.622	0.054
rs4707436	88851751	A	0.382	0.467	0.030	0.463	0.031	0.538	0.042
rs1049353	88853635	T	0.058	0.629	0.045	0.659	0.037	0.525	0.109
rs806369	88856178	T	0.139	0.193	-0.089	0.173	-0.098	0.597	-0.063
rs806370	88856331	T	0.192	*0.017*	-0.120	*0.023*	-0.110	0.085	-0.151
rs806371	88856363	G	0.331	*0.018*	-0.104	*0.019*	-0.102	*0.029*	-0.155
rs806372	88856563	C	0.192	*0.017*	-0.121	*0.022*	-0.110	0.086	-0.151
rs806373	88856916	A	0.448	0.447	-0.033	0.450	-0.032	0.888	-0.010
rs806374	88857320	C	0.485	0.147	-0.062	0.143	-0.063	0.267	-0.081
rs806376	88858648	T	0.309	0.084	-0.075	0.075	-0.079	0.122	-0.106
rs806378	88859551	T	0.090	0.665	-0.033	0.686	-0.028	0.627	-0.066
rs2023239	88860482	C	0.367	0.534	0.025	0.536	0.025	0.220	0.082
rs1535255	88861208	G	0.273	0.903	0.006	0.902	0.006	0.545	0.048
rs806379	88861267	A	0.471	0.432	-0.033	0.422	-0.034	0.629	-0.034
rs9444584	88862559	C	0.444	*0.023*	-0.091	*0.020*	-0.095	0.092	-0.107
rs806380	88864653	G	0.191	0.310	-0.057	0.335	-0.051	0.122	-0.139
rs806381	88865901	G	0.330	0.158	0.067	0.163	0.065	*0.032*	0.164
rs12528858	88867488	G	0.025	0.785	-0.035	0.810	-0.027	0.335	0.245
rs12205430	88867925	C	0.053	0.301	-0.106	0.336	-0.091	0.240	-0.222
rs6933128	88868830	A	0.165	0.774	0.018	0.775	0.018	0.803	0.024
rs6454673	88871049	A	0.313	0.212	0.060	0.208	0.062	0.142	0.113

rs9353526	88876576	T	0.256	0.060	-0.094	0.059	-0.094	0.079	-0.139
rs9362466	88876581	T	0.258	0.072	-0.089	0.071	-0.090	0.067	-0.144
rs2180619	88877952	A	0.256	0.080	-0.087	0.079	-0.088	0.091	-0.133

Notes: (1) MAF = minor allele frequency; (2) Nominal significances given in italic ($p < 0.05$); (3) Bonferroni correction significances given in bold ($p < 1.9E-03$).

Supplemental Table S5: Statistical results for association analysis with Agreeableness

SNP ID	Position	Minor allele	MAF	SNPTEST				PLINK	
				Expected P	Expected Beta	Score P	Score Beta	Plink P	Plink Beta
rs806366	88847589	C	0.186	0.652	-0.026	0.658	-0.025	0.912	0.016
rs806368	88850100	C	0.210	0.155	-0.069	0.185	-0.060	*0.025*	-0.266
rs12720071	88851181	C	0.151	0.660	-0.027	0.657	-0.027	0.901	0.020
rs4707436	88851751	A	0.382	0.212	0.051	0.219	0.050	0.350	0.091
rs1049353	88853635	T	0.058	0.422	0.074	0.468	0.060	0.463	0.182
rs806369	88856178	T	0.139	0.426	-0.054	0.424	-0.055	0.524	0.110
rs806370	88856331	T	0.192	0.256	-0.057	0.286	-0.050	0.052	-0.245
rs806371	88856363	G	0.331	0.645	-0.020	0.656	-0.019	0.676	-0.043
rs806372	88856563	C	0.192	0.256	-0.057	0.285	-0.050	0.055	-0.242
rs806373	88856916	A	0.448	0.871	0.007	0.874	0.007	0.442	0.080
rs806374	88857320	C	0.485	0.716	-0.016	0.721	-0.015	0.700	-0.040
rs806376	88858648	T	0.309	0.695	-0.017	0.704	-0.016	0.346	-0.096
rs806378	88859551	T	0.090	0.678	0.031	0.700	0.027	0.421	0.159
rs2023239	88860482	C	0.367	0.783	0.011	0.786	0.011	0.261	0.110
rs1535255	88861208	G	0.273	0.861	-0.008	0.862	-0.008	0.894	-0.015
rs806379	88861267	A	0.471	0.703	-0.016	0.710	-0.015	0.160	-0.140
rs9444584	88862559	C	0.444	0.805	-0.010	0.809	-0.009	0.332	-0.090
rs806380	88864653	G	0.191	0.558	-0.033	0.571	-0.030	0.251	-0.149
rs806381	88865901	G	0.330	0.783	0.013	0.786	0.013	0.499	0.074
rs12528858	88867488	G	0.025	0.190	0.170	0.227	0.144	0.087	0.630
rs12205430	88867925	C	0.053	0.306	-0.104	0.344	-0.089	0.772	-0.079

rs6933128	88868830	A	0.165	0.845	-0.012	0.841	-0.013	0.906	0.017
rs6454673	88871049	A	0.313	0.563	0.028	0.564	0.028	0.298	0.115
rs9353526	88876576	T	0.256	0.058	-0.094	0.072	-0.084	*0.018*	-0.272
rs9362466	88876581	T	0.258	0.052	-0.096	0.066	-0.086	*0.019*	-0.269
rs2180619	88877952	A	0.256	*0.045*	-0.100	0.058	-0.089	*0.007*	-0.311

Notes: (1) MAF = minor allele frequency; P = P value; (2) Nominal significances given in italic ($p < 0.05$); (3) Bonferroni correction significances given in bold ($p < 1.9E-03$).

Supplemental Table S6: Statistical results for association analysis with Neuroticism

SNP ID	Position	Minor allele	MAF	SNPTEST				PLINK	
				Expected P	Expected Beta	Score P	Score Beta	Plink P	Plink Beta
rs806366	88847589	C	0.186	0.921	0.006	0.920	0.006	0.553	-0.081
rs806368	88850100	C	0.210	0.484	-0.034	0.497	-0.032	0.587	-0.061
rs12720071	88851181	C	0.151	0.444	0.046	0.440	0.047	0.811	-0.036
rs4707436	88851751	A	0.382	0.503	-0.028	0.508	-0.027	0.130	-0.142
rs1049353	88853635	T	0.058	0.379	0.081	0.406	0.072	0.746	0.077
rs806369	88856178	T	0.139	0.611	0.035	0.597	0.038	0.940	0.012
rs806370	88856331	T	0.192	0.402	-0.042	0.412	-0.041	0.468	-0.087
rs806371	88856363	G	0.331	0.619	-0.022	0.624	-0.021	0.730	-0.034
rs806372	88856563	C	0.192	0.401	-0.043	0.410	-0.041	0.480	-0.085
rs806373	88856916	A	0.448	0.880	0.006	0.881	0.006	0.372	0.089
rs806374	88857320	C	0.485	0.711	0.016	0.706	0.016	0.569	0.057
rs806376	88858648	T	0.309	0.849	0.008	0.852	0.008	0.699	0.038
rs806378	88859551	T	0.090	0.514	-0.049	0.514	-0.049	0.801	-0.047
rs2023239	88860482	C	0.367	0.941	0.003	0.940	0.003	0.694	0.036
rs1535255	88861208	G	0.273	0.717	-0.017	0.714	-0.018	0.879	-0.017
rs806379	88861267	A	0.471	0.874	0.007	0.875	0.007	0.749	-0.030
rs9444584	88862559	C	0.444	0.793	0.010	0.795	0.010	0.940	0.007
rs806380	88864653	G	0.191	0.745	0.018	0.745	0.018	0.993	-0.001
rs806381	88865901	G	0.330	0.905	0.006	0.903	0.006	0.647	-0.047

rs12528858	88867488	G	0.025	0.566	-0.075	0.572	-0.072	0.410	-0.290
rs12205430	88867925	C	0.053	0.769	-0.030	0.766	-0.031	0.893	0.035
rs6933128	88868830	A	0.165	0.876	-0.010	0.871	-0.011	0.964	0.006
rs6454673	88871049	A	0.313	0.834	-0.010	0.830	-0.011	0.758	0.032
rs9353526	88876576	T	0.256	0.309	0.051	0.320	0.048	0.084	0.189
rs9362466	88876581	T	0.258	0.342	0.047	0.351	0.045	0.085	0.189
rs2180619	88877952	A	0.256	0.266	0.055	0.279	0.052	0.106	0.178

Notes: (1)MAF = minor allele frequency; (2) Nominal significances given in italic (p < 0.05); (3) Bonferroni correction significances given in bold (p < 1.9E-03).

Supplemental Table S7: Identified SNPs associated with five dimensions of personality traits

SNP ID	Position	Minor allele	MAF	Extraversion P-value	Conscientiousness P-value	Openness P-value	Agreeableness P-value	Neuroticism P-value	literature evidence	SNP priority score
rs806368	88850100	C	0.210	*0.002*	0.294	*0.021*	0.155	0.484	Ehlers *et al*	5
rs12720071	88851181	C	0.151	*0.028*	0.073	0.466	0.660	0.444	NULL	2
rs806369	88856178	T	0.139	*0.016*	*0.013*	0.193	0.426	0.611	NULL	2
rs806370	88856331	T	0.192	**2.68E-04**	0.150	*0.017*	0.256	0.402	NULL	2
rs806371	88856363	G	0.331	*0.013*	*0.044*	*0.018*	0.645	0.619	Mitjans *et al*	4
rs806372	88856563	C	0.192	**2.66E-04**	0.151	*0.017*	0.256	0.401	NULL	2
rs806376	88858648	T	0.309	*0.002*	0.174	0.084	0.695	0.849	NULL	1
rs806379	88861267	A	0.471	0.037	0.851	0.432	0.703	0.874	Juhasz *et al*	2
rs9444584	88862559	C	0.444	0.007	0.351	*0.023*	0.805	0.793	NULL	2
rs806381	88865901	G	0.330	0.135	0.330	0.158	0.783	0.905	NULL	1
rs6454673	88871049	A	0.313	0.032	0.256	0.212	0.563	0.834	NULL	1
rs9353526	88876576	T	0.256	*0.002*	**9.97E-04**	0.060	0.058	0.309	NULL	3
rs9362466	88876581	T	0.258	*0.003*	**1.36E-03**	0.072	0.052	0.342	NULL	3
rs2180619	88877952	A	0.256	**1.53E-03**	**3.01E-04**	0.080	*0.045*	0.266	Heitland *et al*	5

Notes: (1) MAF = minor allele frequency; (2) Nominal significances given in italic (p < 0.05); (3) Bonferroni correction significances given in bold (p < 1.9E-03).

Supplemental Table S8: Allele frequency comparison among our African-American population, 1000Genome African population and 1000Genome European population.

SNP ID	Position	Allele	African-American	1000G AFR	1000G EUR
rs806366	88847589	C	0.186	0.208	0.513
rs806368	88850100	C	0.210	0.076	0.218
rs12720071	88851181	C	0.151	0.177	0.098
rs4707436	88851751	A	0.382	0.417	0.264
rs1049353	88853635	T	0.058	0.029	0.258
rs806369	88856178	T	0.139	0.124	0.312
rs2023239	88860482	C	0.367	0.368	0.157
rs1535255	88861208	G	0.273	0.372	0.157
rs806379	88861267	A	0.471	0.442	0.554

Note: (1) 1000G AFR=1000Genome African population; (2)1000G EUR=1000Genome European population.
